# Coexistence of Substance Abuse among Emergency Department Patients Presenting with Suicidal Ideation

**DOI:** 10.1155/2020/7460701

**Published:** 2020-09-29

**Authors:** Allison Tadros, Melinda Sharon, Michael Crum, Ryan Johnson, Kimberly Quedado, Wei Fang

**Affiliations:** ^1^West Virginia University Department of Emergency Medicine, USA; ^2^West Virginia Clinical & Translational Science Institute, USA

## Abstract

**Background:**

Patients who are suicidal commonly seek care in the emergency department (ED). Few studies have examined the coexistence between suicidal ideation, substance abuse, and psychiatric diagnosis.

**Objectives:**

This study sought to determine how often suicidal ED patients have coexisting substance abuse and psychiatric diagnosis in addition to describing the characteristics of target population.

**Methods:**

In this retrospective cohort study, chart reviews were conducted of patients over 12 with suicidal ideation who presented to an academic ED from October 2016 to March 2017. Data abstracted included gender, age, insurance, prior psychiatric diagnoses, substances abused, presence of a suicide attempt, prior suicidality, number of ED visits in the prior year, and disposition. Both descriptive and inferential statistics were calculated.

**Results:**

There were 427 patient visits to the ED for suicidality during the study period, of which 54% were male, with a mean age of 34 years. Most patients (92%) had a psychiatric diagnosis, most commonly depression (67%). More than one psychiatric diagnosis was reported in 51% of patients, while 8% had no reported underlying psychiatric diagnosis. Substance abuse was reported in 58% of patients, including marijuana (42%) and opioids (41%). Polysubstance abuse was reported in 42%. Approximately half of the patients had three or more ED visits in the previous 12 months. Most patients were insured by Medicaid (51%), while 59% were admitted for inpatient treatment.

**Conclusion:**

Substance abuse and psychiatric diagnosis were reported frequently among patients presenting to the ED with suicidal ideation, often involving more than one substance/diagnosis. Future studies should be aimed at evaluating the relationship between these conditions and determining how to better care for this population.

## 1. Introduction

Suicide deaths increased by more than 30% from 1999 to 2017 and now represent the 10th leading cause of death in the United States (US) [1, 2]. Suicide rates have increased in every age group under 75 years of age, with rural counties seeing much greater increases than urban areas [1]. Similarly, deaths attributable to drug overdose have dramatically increased during this century. From the same time period of 1999-2017, overdose deaths from prescription opioids increased five times [3]. Similarly, deaths from drug overdoses rates also have increased more dramatically in rural areas [4].

Recent studies have suggested that approximately 4% of adults in the US have suicidal thoughts annually [5, 6]. Patients with suicidal ideation or suicide attempt commonly present to the emergency department (ED) for evaluation, treatment, and possible placement into a psychiatric facility [7, 8]. The number of patients presenting to the ED for suicidal thoughts has recently increased [8]. In 2013, patients with suicidal ideation represented 1% of all ED visits [8].

West Virginia has seen particularly high rates of substance abuse in the past decade and currently has the highest death rate from drug overdoses in the US at 58 per 100,000 residents [9–11]. West Virginia also has high suicide mortality rates, ranking 8th highest in the country [12]. Despite rising rates of substance abuse, overdose deaths, and suicidal ideation, few studies have examined the coexistence between substance abuse, psychiatric diagnoses, and suicidality. The purpose of this study was to determine how often patients seeking assistance in a West Virginia ED for suicidal ideation or suicide attempt have coexisting substance abuse. In addition, the specific substances being abused were determined. Comparisons were made with substance abuse and psychiatric diagnoses between males and females.

## 2. Materials and Methods

### 2.1. Study Design, Setting, and Patient Selection

This study was conducted at a tertiary care academic ED with an annual census of approximately 50,000 visits. Both pediatric and adult patients are cared for at this facility. We performed a retrospective cohort study of patients aged 13 years and older who visited our ED between October 1, 2016, and March 31, 2017, who were diagnosed by ICD-10 codes with either R45.851 (suicidal ideation) or T14.91 (suicide attempt).

### 2.2. Data Collection and Analyses

The charts of patients meeting the inclusion criteria were reviewed by 3 of the authors (AT, RJ, and MC). Data abstracted included age, gender, number of ED visits in the prior 12 months, current substances being used at the time of visit, insurance status, disposition, whether the patient self-reported any prior suicide attempt(s), previously diagnosed psychiatric disorders, and if they reported a suicide attempt immediately preceding the current visit. One reviewer (AT) reviewed approximately 50% of the other reviewers' charts for consistency purposes.

Descriptive statistics regarding age, gender, substances, psychiatric diagnoses, number of ED visits, and disposition were calculated. Regarding the frequency of substance use or psychiatric diagnosis, if a patient was indicated to use more than one substance, they were listed in a polysubstance category; otherwise, their case was limited to the single substance or no substance; synonymously, if a patient had more than one psychiatric diagnosis, they were separated into a multiple diagnosis category. If there was one or no diagnosis, they remained in their specific diagnosis category as well. In addition, Fisher's exact test was conducted to determine whether there is a significant difference in the distribution of either single substance abuse or polysubstance abuse cases between males and females and whether there is a significant difference in the distribution of either single psychiatric diagnosis or multiple psychiatric diagnoses between males and females. All statistical analysis was conducted using SAS 9.4 (The SAS Institute, Cary, NC). This study was approved by the university's Institutional Review Board.

## 3. Results

From October 1, 2016, to March 31, 2017, there were 427 patients seen in the ED with suicidal ideation or suicide attempt. Males represented 53% of the total sample, with an average age of 34 years, ranging from 13 to 84 years. Medicaid was the primary insurer for 51% of the sample.

Overall, 51% of the patients were admitted to a psychiatric facility or the hospital (Table 1).

In addition to suicidal ideation or attempt, 59% of the patients also reported abuse of at least one substance. Substance abuse was reported in 56% of males and 46% of females. The most commonly abused substances in males were opioids and marijuana, while 28% reported polysubstance abuse (Table 2). Females most commonly abused opioids and marijuana as well, with 21% reporting the abuse of more than one substance (Table 2). Of patients with suicidal ideation or attempt who had either no substance abuse or abused only one substance, there was no statistical difference in the distribution of substance which was being used between men and women (*p* value = 0.1595). Likewise, there were no gender differences found in the distribution of substances being abused in patients with polysubstance abuse (*p* value = 0.5470).

Underlying psychiatric disorder(s) were common among patients with suicidal ideation or attempt but was not present in all patients (Tables 1 and 3). Depression and anxiety were the most commonly reported psychiatric diagnoses in both men and women, with 47% of men and 49% of women suffered from more than one disorder (Table 3). Only 8% of the patients with suicidal ideation did not have an underlying psychiatric diagnosis, while 5% of the patients reported no substance abuse or underlying psychiatric diagnosis. Further information on underlying psychiatric disorders among the patients presenting with suicidal ideation or attempt can be seen in Table 3. Of the patients with no psychiatric diagnosis or only one psychiatric diagnosis, there was no statistically significant difference in the distribution or diagnosis between men and women (*p* value = 0.1170).

Several patients included in the study had made more than one visit in the prior 12 months to the same ED. Frequent visits were particularly common in the older age groups (Table 4). Although patients over 50 years of age comprised only 19% of the total population of visits for suicidal ideation, 24% of them had over 10 visits in a year.

## 4. Discussion

Patients with substance abuse and suicidal ideation are both commonly encountered in EDs [13–15]. Mental health and substance abuse disorders account for approximately one out of every eight ED visits in the US and are commonly encountered in the same patient populations [15, 16]. Suicide represents a growing public health concern and is currently the 10^th^ leading cause of death in WV [11]. Suicide is also the leading cause of death among people with substance use disorders [17]. The relationship between substance abuse and increased risk of suicide is well demonstrated in both the psychiatric and public health literatures. Given the prevalence of these diagnoses in ED patients, this study attempted to quantify the frequency of substance abuse in patients with suicidal ideation or attempt.

This study found that substance abuse was relatively common in ED patients presenting with suicidal ideation, with over half of the patients with suicidal ideation in the ED also reporting substance abuse. The most commonly abused substances among both men and women were marijuana and opioids, while over 20% of patients abused more than one substance. Of note, this study was conducted in a state where marijuana use is currently illegal. The percentage of patients self-reporting only alcohol use was relatively low at 17%, which is possibly the result of patients using alcohol in addition to other substances [18]. Although prior studies have found gender differences in substance abuse, statistically significant differences were not found within our patient cohort [19, 20].

In 2015, there were an estimated 5.7 million ED visits in the US, with the primary diagnosis of a psychiatric disorder [21]. In the current study, it is not surprising that the vast majority had at least one existing psychiatric diagnosis. Mood disorders were most commonly seen, with 67% reporting depression and 40% suffering from anxiety. Roughly half of the study population had multiple psychiatric diagnoses, and over one-third of patients had a history of at least one prior suicide attempt. Other studies have also found frequent recurrence of suicidal ideation and attempts, with a higher likelihood in patients with concomitant substance abuse [22, 23]. This data highlights the need for ongoing care and the potentially recurrent nature of psychiatric illness in patients with suicidal thoughts and substance abuse.

Almost half of the patients presenting for suicidal ideation had three or more visits to the same ED within the previous 12 months, and there were many patients who had over 10 visits in the prior year. These encounters only include visits to our facility and do not account for possible visits to other facilities. A previous study found that 14% of the patients with an ED visit for mental health or substance abuse had a return visit to the ED or hospitalization within 30 days, particularly for older adults [22]. Other studies have similarly reported that patients with both a psychiatric and substance abuse diagnoses had significantly higher ED utilization [14, 24–26]. These findings highlight the chronic nature of substance abuse and psychiatric illness and therefore the need for adequately resourced psychiatric care programs.

Males were more commonly seen in our ED for suicidal ideation than females. This is in contrast to an earlier study which found a slight female prevalence [27]. Medicaid was the payer for over half of the visits in this study, followed by private insurance. A study examining ED visits nationally for suicidal ideation reported that Medicaid was the insurer for 28% of patients, with over 25% of these patients being uninsured [27]. The present study had nearly double the amount of patients on Medicaid as compared to this brief report. However, that study was conducted from 2006-2013 and therefore included data prior to the Affordable Care Act.

Although the majority of patients in the present study were admitted to an inpatient treatment setting, a substantial minority of patients (40%) were discharged from the ED with a plan to establish or continue existing outpatient treatment. In comparison, a national study reported that 27% of this patient population was discharged upon their ED visit [27]. It is worth further examining in future research what factors contribute to this patient population's disposition and how suicidal ideation and/or substance abuse affects this decision.

### 4.1. Limitations

There are several limitations to this study that warrant further discussion. First, this study was conducted at a single ED, limiting the diversity of the studied patient population, and potentially limits the generalizability. Factors such as the disproportionately higher rates of opioid abuse among this state population when compared to the national rate may contribute to this [10]. Second, this study was a retrospective chart review and was subjected to the inherent limitations of this type of study, including potential selection, recall, and misclassification biases. Another potential limitation was variability in data collection due to factors including inconsistencies of the electronic medical record, variability in provider documentation, and variation between data abstracter methods. Lastly, another important limitation was the reliance on either patient self-reporting or the use of potentially inaccurate urine drug screen results to classify patient substance abuse status.

## 5. Conclusions

This study further illustrated that substance abuse is commonly encountered in emergency department patients with suicidal ideation. Polysubstance abuse and suffering from multiple psychiatric diagnoses were also relatively common. Caring for these patients is complex and multifaceted. Further research is needed to analyze the relationship between suicidality, gender, substance abuse, and mental health as well as how to best serve this population. In particular, studies to determine if the treatment of substance abuse leads to a reduction in suicidal ideation and attempt would be useful to practitioners.

## Figures and Tables

**Table 1:** Demographic characteristics of patients presenting to the emergency department (ED) for suicidality.

Characteristic	*N* (%)
Total	427
Gender	
Male	228 (53)
Female	199 (47)
Age (years)	
Average	34
Range	13–84
Payor type	
Medicare	59 (14)
Medicaid	216 (51)
Private	142 (33)
Self-pay	6 (1.1)
Uninsured	3 (0.7)
Other	1 (0.2)
Coexisting substance abuse	
Yes	250 (59)
No	177 (41)
Underlying psychiatric diagnosis	
Yes	392 (92)
No	35 (8)
ED disposition	
Admit	253 (59)
Discharge	173 (40)
Eloped	1 (1)

**Table 2:** Gender comparisons of self-reported substances. Substances may be reported both individually and under polysubstance if applicable.

	Males (%) *N* = 228	Females (%) *N* = 199
Alcohol	35 (15)	11 (6)
Amphetamines	29 (13)	23 (12)
Barbiturates	1 (0.4)	0 (0)
Bath salts	0 (0)	1 (0.5)
Benzo	30 (13)	24 (12)
Buprenorphine	0 (0)	1 (0.5)
Cocaine	3 (1.3)	1 (0.5)
LSD	1 (0.4)	1 (0.5)
Marijuana	63 (28)	43 (22)
Opioid	59 (26)	47 (24)
Polysubstances	63 (28)	42 (21)
None	100 (44)	107 (54)

**Table 3:** Gender comparisons of psychiatric diagnoses. Diagnoses may be reported both individually and under multiple diagnoses. (Note: males *n* = 228, females *n* = 199).

	Male (%)	Female (%)
ADHD	25 (11)	16 (8.0)
Anxiety	69 (30)	92 (46)
Bipolar	27 (12)	15 (7.5)
Depression	136 (60)	132 (66)
OCD	0 (0)	1 (0.5)
ODD	2 (0.9)	0 (0)
PTSD	37 (16)	41 (21)
Thought disorder	36 (16)	9 (4.5)
Multiple diagnoses	108 (47)	97 (49)
None	18 (7.9)	16 (8)

**Table 4:** Number of ED visits in the past six months by patients grouped by age (in years).

	1 to 2 (%)	3 to 4 (%)	5 to 6 (%)	7 to 10 (%)	More than 10 (%)	Total (%)
Less than 18	51 (77)	10 (15)	4 (6.5)	1 (1.5)	0 (0)	66 (15)
18 to 29	80 (57)	22 (16)	16 (11)	9 (6.1)	14 (9.9)	141 (33)
30 to 49	63 (45)	20 (14)	10 (7.1)	11 (7.9)	36 (26)	140 (33)
50 and older	37 (47)	16 (20)	5 (6.5)	2 (2.5)	19 (24)	79 (19)

## Data Availability

The collection of the data for this research paper required the authors to enter patient medical records, with approval of our Institutional Review Board. This data would therefore only be available to the authors.
